# Advancing cancer treatments: The role of oligonucleotide-based therapies in driving progress

**DOI:** 10.1016/j.omtn.2024.102256

**Published:** 2024-06-17

**Authors:** Bogdan Dume, Emilia Licarete, Manuela Banciu

**Affiliations:** 1 Doctoral School in Integrative Biology, Faculty of Biology and Geology, Babes-Bolyai University, 400006 Cluj-Napoca, Romania; 2 Department of Molecular Biology and Biotechnology, Centre of Systems Biology, Biodiversity and Bioresources, Faculty of Biology and Geology, Babes-Bolyai University, 400006 Cluj-Napoca, Romania

**Keywords:** MT: Oligonucleotides: Therapies and Applications, oligonucleotides, cancer therapy, delivery systems, clinical trials, tumor targeting

## Abstract

Although recent advancements in cancer immunology have resulted in the approval of numerous immunotherapies, minimal progress has been observed in addressing hard-to-treat cancers. In this context, therapeutic oligonucleotides, including interfering RNAs, antisense oligonucleotides, aptamers, and DNAzymes, have gained a central role in cancer therapeutic approaches due to their capacity to regulate gene expression and protein function with reduced toxicity compared with conventional chemotherapeutics. Nevertheless, systemic administration of naked oligonucleotides faces many extra- and intracellular challenges that can be overcome by using effective delivery systems. Thus, viral and non-viral carriers can improve oligonucleotide stability and intracellular uptake, enhance tumor accumulation, and increase the probability of endosomal escape while minimizing other adverse effects. Therefore, gaining more insight into fundamental mechanisms of actions of various oligonucleotides and the challenges posed by naked oligonucleotide administration, this article provides a comprehensive review of the recent progress on oligonucleotide delivery systems and an overview of completed and ongoing cancer clinical trials that can shape future oncological treatments.

## Graphical abstract

**Figure:**
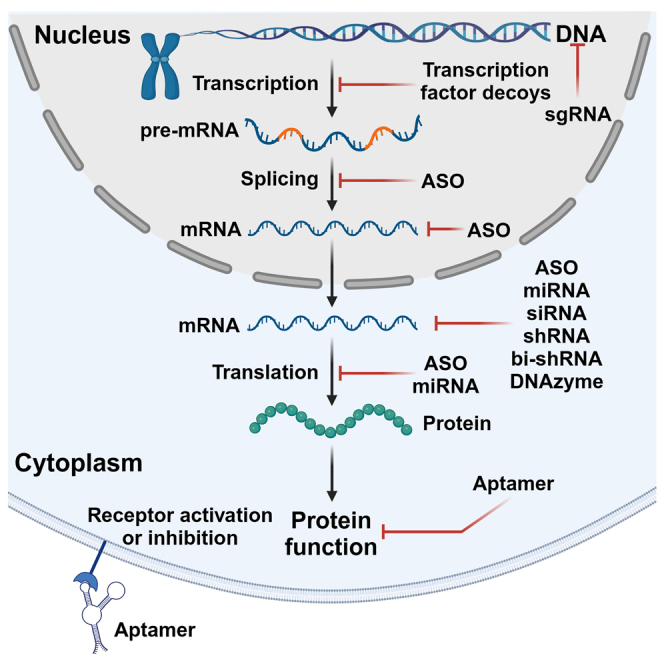

## Teaser

Licarete and colleagues offer an updated review regarding the advances in the oligonucleotides used in cancer therapy. They describe the main classes of oligonucleotides, their mechanisms of action, as well as different delivery systems. They also provide an updated overview of completed and ongoing oligonucleotide-based clinical trials in cancer.

## Introduction

Up to 10 million deaths and 19.3 million newly diagnosed cancer cases were reported worldwide in 2020, according to the World Health Organization^1^ and recent statistical data have suggested that the global incidence of cancer is expected to double in the coming decades.^2^ Nevertheless, significant advancements in early detection methods and surgical procedures have fueled progress in the battle against cancer.^3^ Over the last 20 years, oncological research has been focused on addressing the limitations of traditional treatments such as drug resistance and cancer recurrence. Therefore, there has been a remarkable paradigm shift in cancer treatment, transitioning from treatment strategies based on broad-spectrum cytotoxic drugs to targeted therapies. Unlike conventional chemotherapeutics, targeted drugs exhibit the ability to selectively recognize cancer cells while preserving normal cells, resulting in potent efficacy and minimal toxicity.^4^^,^^5^

**Figure 1:**
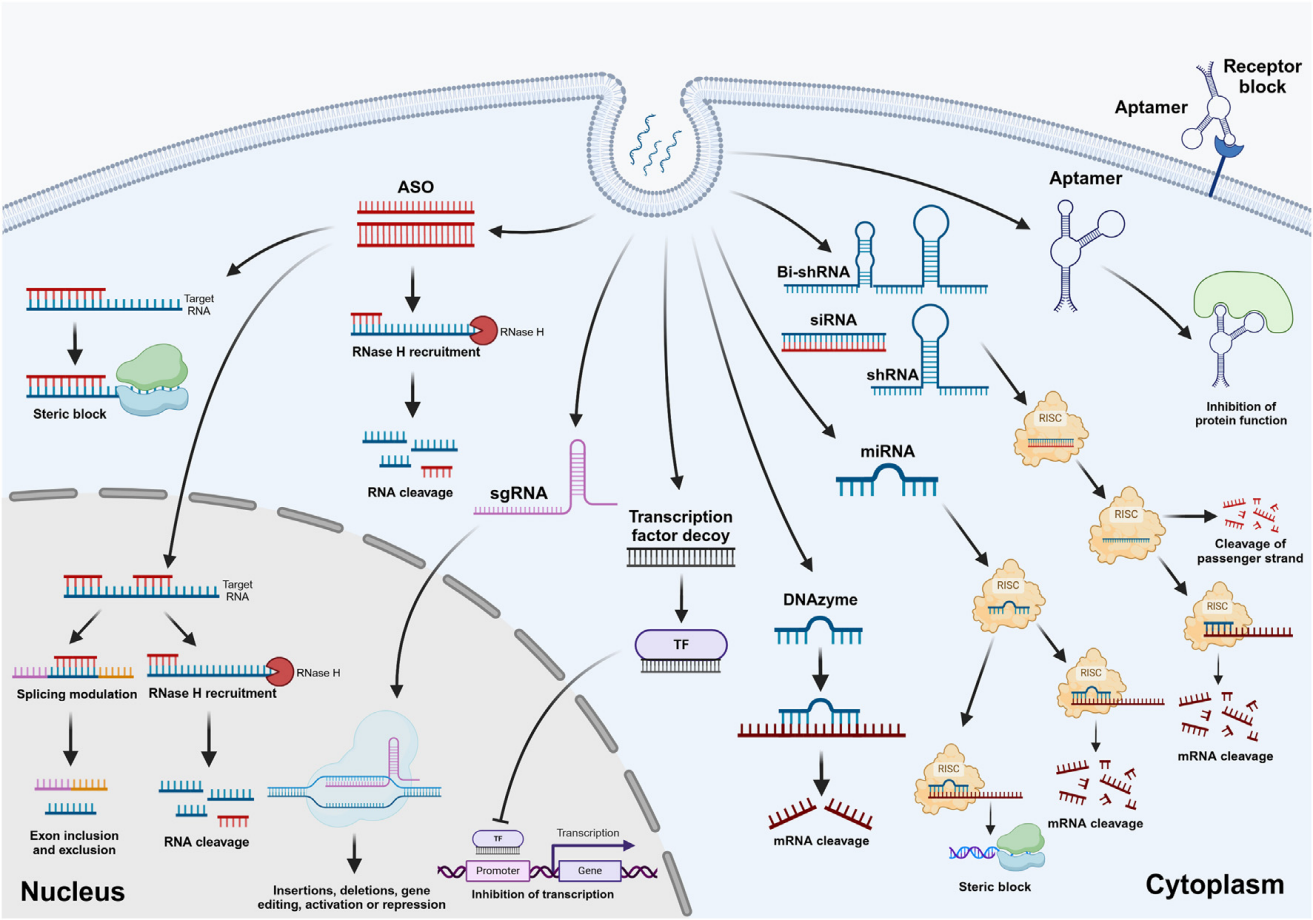
Standard schematic representation of ON activity as cancer therapeutics Antisense ONs (ASOs) can physically obstruct or impede the splicing process. sgRNA guides the Cas9 endonuclease toward creating a double-stranded break at a designated position within the genome, favoring deletions and insertions. Transcription factor decoys bind to transcription factors of targeted DNA during the initial stage, blocking their activity. ASOs, gapmers, small interfering RNAs (siRNAs), microRNAs (miRNAs), short hairpin RNAs (shRNAs), bifunctional short hairpin RNAs (bi-shRNAs), and DNAzymes act to target pre-mRNAs/mRNAs to downregulate or block the production of proteins. Aptamers directly inhibit proteins involved in pathogenesis. Created with BioRender.com.

Since understanding of the molecular mechanisms of tumor development is progressively advancing, and numerous molecular targets have been identified, oligonucleotides (ONs) such as antisense ONs (ASOs), RNA interference (RNAi) molecules, aptamers, DNAzymes, and transcription factor decoys (TFDs) addressed different therapeutic applications across cancer. Thus, ONs, due to their various mechanisms of action, including gene silencing,^6^ splice modulation,^7^ and protein interaction,^8^ offer a versatile platform for oncological drug development. Nevertheless, the clinical development of naked ON therapeutics faced challenges given by their physico-chemical properties (size, charge), off-target effects, interactions with the immune system, rapid clearance, and nuclease degradation. Therefore, the tumor-targeted delivery of these agents was proposed as an attractive strategy for their oncological application.^9^^,^^10^^,^^11^^,^^12^ Thus, in tight connection with recent findings, this review brought insight into the oncological treatment approaches based on different types of ONs and their mechanisms of action (Figure 1; Table 1). Moreover, the second part of the review presented the most relevant delivery systems proposed to improve the tumor delivery of ONs and the current state of clinical trials testing the antitumor efficacy of ON therapeutics (Table 2).

**Table 1:** Therapeutic classes of ONs used in cancer

	Structure	Target	Mechanism
Antisense ON	ssDNA or ssRNA	DNA and RNA (pre-miRNA, mRNA, lncRNA)	RNA cleavage, RNA blockage, or splicing modulation
Gapmer	ssDNA	mRNA	RNA cleavage
miRNA	ssRNA	mRNA	RNA cleavage or translational inhibition
siRNA	dsRNA	mRNA	RNA cleavage
shRNA	ssRNA and dsRNA	mRNA	RNA cleavage
bi-shRNA	ssRNA and dsRNA	mRNA	RNA cleavage and translational inhibition
Aptamer	ssDNA or ssRNA	small molecules, peptides, and proteins	inhibition of function
DNAzyme	ssDNA	ssDNA or RNA	RNA cleavage
Transcription factor decoy	dsDNA	transcription factor	inhibition of transcription
sgRNA	ssRNA	dsDNA	insertions, deletions, gene editing, activation, or repression

miRNA, microRNA; siRNA, small interfering RNA; shRNA, short hairpin RNA; bi-shRNA, bifunctional short hairpin RNA; sgRNA, single guide RNA; ssDNA, single-stranded DNA; dsDNA, double-stranded DNA; ssRNA, single-stranded RNA; dsRNA, double-stranded RNA; pre-miRNA, precursor miRNA; mRNA, messenger RNA; lncRNA, long non-coding RNA.

**Table 2:** Completed, active, or recruiting clinical trials based on ONs therapy for cancer treatment

Completed clinical trials						
Identifier	Disease	Oligonucleotide	Delivery strategies	Target	Phase	Reference
**Antisense ONs**						

NCT00431561	glioblastoma, anaplastic astrocytoma	AP 12009	PS linkages	*TGFβ2*	II	Uckun et al.^13^
NCT00844064	pancreatic and colorectal neoplasms, melanoma	AP 12009	PS linkages	*TGFβ2*	I	
NCT00074737	acute myelogenous leukemia	cenersen	PS linkages	*TP53*	II	
NCT00002592	leukemia	G4460	PS linkages	*C-MYB*	II	
NCT00780052	hematologic malignancies	G4460	PS linkages	*C-MYB*	I	
NCT00056173	carcinoma, renal cell, metastases, neoplasm	GTI-2040	PS linkages	R2 subunit of RNR	I/II	
NCT00565058	acute myeloid leukemia	GTI-2040	PS linkages	R2 subunit of RNR	II	
NCT00068588	male breast cancer, recurrent breast cancer, stage IV breast cancer	GTI-2040	PS linkages	R2 subunit of RNR	II	
NCT00087165	prostate cancer	GTI-2040	PS linkages	R2 subunit of RNR	II	Sridhar et al.^14^
NCT00005594	pancreatic cancer	ISIS 2503	PS linkages	*HRAS*	II	
NCT00006467	pancreatic cancer	ISIS 2503	PS linkages	*HRAS*	II	Alberts et al.^15^
NCT00017407	lung cancer	ISIS 3521	PS linkages	*PKCA*	III	
NCT00003989	lung cancer, melanoma	ISIS 3521	PS linkages	*PKCA*	II	
NCT00034268	carcinoma, non-small cell lung	LY900003	PS linkages	*PKCA*	III	
NCT00042679	carcinoma, non-small cell lung	LY900003	PS linkages	*PKCA*	II	
NCT00003892	ovarian cancer	ISIS 5132	PS linkages	*CRAF*	II	Oza et al.^16^
NCT00003236	breast cancer	ISIS 3521 ISIS 5132	PS linkages	*PKCA* *CRAF*	II	
NCT00004862	leukemia	oblimersen sodium	PS linkages	*BCL-2*	I	
NCT00017589	leukemia	oblimersen sodium	PS linkages	*BCL-2*	II	Moore et al.^17^
NCT00024440	leukemia	oblimersen sodium	PS linkages	*BCL-2*	III	O’Brien et al.^18^^,^^19^
NCT00049192	chronic myelogenous leukemia, BCR-ABL1-positive, chronic phase chronic myelogenous leukemia, relapsing chronic myelogenous leukemia	oblimersen sodium	PS linkages	*BCL-2*	II	
NCT00039117	adult acute myeloid leukemia with 11q23 abnormalities, Inv(16)(p13;q22), t(16;16)(p13;q22), (8;21)(q22;q22), secondary acute myeloid leukemia	oblimersen sodium	PS linkages	*BCL-2*	I	
NCT00085124	adult acute myeloid leukemia with 11q23 abnormalities, Inv(16)(p13;q22), t(15;17)(q22;q12), t(16;16)(p13;q22), t(8;21)(q22;q22), secondary acute myeloid leukemia	oblimersen sodium	PS linkages	*BCL-2*	III	Walker et al., Yin et al.^20^^,^^21^^,^^22^
NCT00021749	chronic lymphocytic leukemia	oblimersen sodium	PS linkages	*BCL-2*	I/II	O’Brien et al.^23^
NCT00078234	chronic lymphocytic leukemia	oblimersen sodium	PS linkages	*BCL-2*	I/II	
NCT00049374	multiple myeloma and plasma cell neoplasm	oblimersen sodium	PS linkages	*BCL-2*	II	Badros et al.^24^
NCT00017602	multiple myeloma and plasma cell neoplasm	oblimersen sodium	PS linkages	*BCL-2*	III	Chanan-Khan et al.^25^
NCT00070083	lymphoma	oblimersen sodium	PS linkages	*BCL-2*	I	
NCT00054639	cutaneous B cell non-Hodgkin lymphoma, extranodal marginal zone B cell lymphoma of mucosa-associated lymphoid tissue, intraocular lymphoma, nodal marginal zone B cell lymphoma, recurrent adult Burkitt lymphoma, recurrent adult diffuse large cell lymphoma, recurrent adult diffuse mixed cell lymphoma, recurrent adult diffuse small cleaved cell lymphoma, recurrent adult grade III lymphomatoid granulomatosis, recurrent adult immunoblastic large cell lymphoma, recurrent adult lymphoblastic lymphoma, recurrent grade I, II, III follicular lymphoma, recurrent mantle cell lymphoma, recurrent marginal zone lymphoma, recurrent small lymphocytic lymphoma, small intestine lymphoma, splenic marginal zone lymphoma, testicular lymphoma, Waldenström macroglobulinemia	oblimersen sodium	PS linkages	*BCL-2*	II	
NCT00086944	recurrent adult diffuse large cell lymphoma, recurrent grade 3 follicular lymphoma, recurrent mantle cell lymphoma	oblimersen sodium	PS linkages	*BCL-2*	I/II	
NCT00062244	Waldenström macroglobulinemia	oblimersen sodium	PS linkages	*BCL-2*	I/II	
NCT00005032	lung cancer	oblimersen sodium	PS linkages	*BCL-2*	I/II	Rudin et al.^26^
NCT00017251	extensive stage small cell lung cancer	oblimersen sodium	PS linkages	*BCL-2*	I	
NCT00042978	extensive stage small cell lung cancer, recurrent small cell lung cancer	oblimersen sodium	PS linkages	*BCL-2*	II	
NCT00047229	liver cancer	oblimersen sodium	PS linkages	*BCL-2*	II	Knox et al.^27^
NCT00004870	colorectal cancer	oblimersen sodium	PS linkages	*BCL-2*	I/II	
NCT00055822	colorectal cancer	oblimersen sodium	PS linkages	*BCL-2*	I/II	
NCT00059813	recurrent and stage IV renal cell cancers	oblimersen sodium	PS linkages	*BCL-2*	II	
NCT00085228	prostate cancer	oblimersen sodium	PS linkages	*BCL-2*	II	Sternberg et al.^28^
NCT00079131	recurrent neuroendocrine carcinoma of the skin, stage I, II, III, IV neuroendocrine carcinoma of the skin	oblimersen sodium	PS linkages	*BCL-2*	II	
NCT00016263	melanoma	oblimersen sodium	PS linkages	*BCL-2*	III	
NCT00518895	melanoma	oblimersen sodium	PS linkages	*BCL-2*	III	Bedikian et al.^29^
NCT00542893	advanced melanoma	oblimersen sodium	PS linkages	*BCL-2*	I	
NCT00003103	bladder, breast, colorectal, esophageal, kidney, lung, ovarian, prostate, and head and neck cancers	oblimersen sodium	PS linkages	*BCL-2*	I/II A	
NCT00543231	tumors	oblimersen sodium	PS linkages	*BCL-2*	I	
NCT00636545	solid tumors	oblimersen sodium	PS linkages	*BCL-2*	I	
NCT00054548	unspecified adult solid tumor	oblimersen sodium	PS linkages	*BCL-2*	I	
NCT00039481	unspecified childhood solid tumor	oblimersen sodium	PS linkages	*BCL-2*	I	
NCT00363974	leukemia, myelomonocytic, acute	AEG35156	2′-OMe modifications with PS linkages	*XIAP*	I/II	
NCT00882869	advanced hepatocellular cancer	AEG35156	2′-OMe modifications with PS linkages	*XIAP*	I/II	Lee et al.^30^
NCT00357747	unspecified adult solid tumor	AEG35156	2′-OMe modifications with PS linkages	*XIAP*	I	
NCT00372736	unspecified adult solid tumor	AEG35156	2′-OMe modifications with PS linkages	*XIAP*	I	
NCT00138658	non-small cell lung cancer	custirsen sodium	2′-O-MOE modifications with PS linkages	*CLU*	I/II	Laskin et al.^31^
NCT00258375	breast cancer	custirsen sodium	2′-O-MOE modifications with PS linkages	*CLU*	II	
NCT00258388	prostate cancer	custirsen sodium	2′-O-MOE modifications with PS linkages	*CLU*	II	Chi et al.^32^
NCT00327340	prostate cancer	custirsen sodium	2′-O-MOE modifications with PS linkages	*CLU*	II	Saad et al., Blumenstein et al.^33^^,^^34^
NCT00054106	prostate cancer	custirsen sodium	2′-O-MOE modifications with PS linkages	*CLU*	I	Chi et al.^35^
NCT01578655	prostate cancer	custirsen sodium	2′-O-MOE modifications with PS linkages	*CLU*	III	Beer et al.^36^
NCT00138918	prostate cancer	custirsen sodium	2′-O-MOE modifications with PS linkages	*CLU*	II	
NCT01188187	prostate cancer	custirsen sodium	2′-O-MOE modifications with PS linkages	*CLU*	III	Chi et al., de Liaño et al.^37^^,^^38^
NCT00471432	bladder cancer, breast cancer, kidney cancer, lung cancer, ovarian cancer, prostate cancer	custirsen sodium	2′-O-MOE modifications with PS linkages	*CLU*	I	Chi et al.^39^
NCT01497470	cancer	custirsen sodium	2′-O-MOE modifications with PS linkages	*CLU*	I	
NCT01829113	non-squamous non-small cell lung cancer	OGX-427	2′-O-MOE modifications with PS linkages	*HSP27*	II	
NCT01844817	pancreatic cancer	OGX-427	2′-O-MOE modifications with PS linkages	*HSP27*	II	Ko et al.^40^
NCT01454089	urologic neoplasms, metastatic bladder cancer, urinary tract neoplasms	OGX-427	2′-O-MOE modifications with PS linkages	*HSP27*	II	
NCT01120470	castration-resistant prostate cancer	OGX-427	2′-O-MOE modifications with PS linkages	*HSP27*	II	
NCT01780545	bladder cancer, urothelial cancer	OGX-427	2′-O-MOE modifications with PS linkages	*HSP27*	II	
NCT00487786	neoplasms	OGX-427	2′-O-MOE modifications with PS linkages	*HSP27*	I	Chi et al.^41^
NCT01107444	non-small cell lung cancer	LY2181308	2′-O-MOE modifications with PS linkages	*SURVIVIN*	II	Natale et al.^42^
NCT01675128	colorectal neoplasms, colorectal carcinoma	ISIS 183750	2′-O-MOE modifications with PS linkages	*EIF4E*	I/II	Duffy et al.^43^
NCT01234038	non-small cell lung cancer	ISIS EIF4E Rx	2′-O-MOE modifications with PS linkages	*EIF4E*	I/II	
NCT01234025	castrate-resistant prostate cancer	ISIS EIF4E Rx	2′-O-MOE modifications with PS linkages	*EIF4E*	I/II	
NCT00903708	advanced cancer	LY2275796	2′-O-MOE modifications with PS linkages	*EIF4E*	I	
NCT03101839	non-small cell lung cancer, advanced solid tumors	AZD4785	cEt modifications with PS linkages	*KRAS*	I	
NCT02144051	advanced solid tumors with androgen receptor pathway as a potential factor	AZD5312	cEt modifications with PS linkages	*AR*	I	
NCT02549651	diffuse large B cell lymphoma	AZD9150	cEt modifications with PS linkages	*STAT3*	I	
NCT03527147	non-Hodgkin’s lymphoma, diffuse large B cell lymphoma	AZD9150	cEt modifications with PS linkages	*STAT3*	I	
NCT01839604	advanced adult hepatocellular carcinoma, hepatocellular carcinoma metastatic	AZD9150	cEt modifications with PS linkages	*STAT3*	I	
NCT03394144	advanced solid malignancies	AZD9150	cEt modifications with PS linkages	*STAT3*	I	Nishina et al.^44^
NCT01563302	advanced cancers, diffuse large B cell lymphoma	IONIS-STAT3Rx	cEt modifications with PS linkages	*STAT3*	I/II	Reilley et al.^45^
NCT04659096	advanced solid tumors	ION537	cEt and 2′-MOE modifications with PS linkages	*YAP1*	I	
NCT02580552	cutaneous T cell lymphoma, mycosis fungoides, chronic lymphocytic leukemia, diffuse large B cell lymphoma ABC subtype	Cobomarsen	LNA with PS linkages	*miR-155*	I	
NCT00466583	carcinoma, lymphoma	EZN-2968	LNA with PS linkages	*HIF-1A*	I	
NCT01120288	neoplasms, liver metastases	EZN-2968	LNA with PS linkages	*HIF-1A*	I	
NCT02564614	carcinoma, hepatocellular	RO7070179	LNA with PS linkages	*HIF-1A*	I	
NCT00285103	chronic lymphocytic leukemia	SPC2996	LNA with PS linkages	*BCL-2*	I/II	Dürig et al.^46^
NCT00024648	neoplasms	LErafAON	liposomes	*CRAF*	I	
NCT00024661	neoplasms	LErafAON	liposomes	*CRAF*	I	
NCT00100672	neoplasms	LErafAON-ETU	liposomes	*CRAF*	I	
NCT00009841	head and neck cancer	EGFR antisense DNA	cationic liposomes	*EGFR*	I	
NCT01592721	squamous cell carcinoma, head and neck cancer	EGFR antisense DNA	cationic liposomes	*EGFR*	I/II	
NCT01159028	recurrent adult acute myeloid leukemia, acute lymphoblastic leukemia, myelodysplastic syndrome	BP1001	liposomes	*GRB2*	I	Ohanian et al.^47^
NCT01191775	lymphoma, prostate cancer, melanoma	PNT2258	liposomes	*BCL-2*	I	Tolcher et al.^48^
NCT01733238	lymphoma, non-Hodgkin’s	PNT2258	liposomes	*BCL-2*	II	Harb et al.^49^
NCT02226965	lymphoma, diffuse large B cell	PNT2258	liposomes	*BCL-2*	II	

**RNAi molecules**						

NCT02369198	malignant pleural mesothelioma, non-small cell lung cancer	miRNA – TargomiRs	EnGeneIC Dream Vectors (EDV nanocells)	multiple oncogenes	I	van Zandwijk et al.^50^
NCT00882180	solid tumors	siRNA-ALN-VSP02	SNALP	*VEGF* *KSP*	I	Tabernero et al.^51^
NCT01158079	solid tumors	siRNA-ALN-VSP02	SNALP	*VEGF* *KSP*	I	Tabernero et al.^51^
NCT02191878	hepatocellular carcinoma, hepatoma, liver cancer, adult; liver cell carcinoma, adult	siRNA-TKM-080301	SNALP	*PLK1*	I/II	El Dika et al.^52^
NCT01262235	neuroendocrine tumors, adrenocortical carcinoma	siRNA-TKM-080301	SNALP	*PLK1*	I/II	
NCT01437007	colorectal, pancreas, gastric, breast, and ovarian cancers with hepatic metastases	siRNA-TKM-080301	SNALP	*PLK1*	I	
NCT04169711	clear cell renal cell carcinoma	siRNA-ARO-HIF2	2′-OMe-modified purines and 2′-F-modified pyrimidines with PS linkages	*HIF-2A*	I	
NCT01808638	carcinoma, pancreatic ductal	siRNA-Atu027	liposomes (AtuPlex)	*PKN3*	I/II	Schultheis et al.^53^
NCT00938574	advanced solid tumors	siRNA-Atu027	liposomes (AtuPlex)	*PKN3*	I	Schultheis et al.^54^
NCT03020017	gliosarcoma; recurrent glioblastoma	siRNA-NU-0129	gold nanoparticle SNA	*BCL-2L12*	I	Kumthekar et al.^55^
NCT01188785	pancreatic ductal adenocarcinoma, pancreatic cancer	siRNA-siG12D LODER	polymeric matrix	*KRASG12D*	I	Golan et al.^56^
NCT04293679	Bowen’s disease cutaneous squamous cell carcinoma *in situ*	siRNA-STP705	polypeptide nanoparticle	*TGF-β1* *COX-2*	I/II	
NCT02736565	Ewing’s sarcoma, Ewing’s tumor metastatic, Ewing’s sarcoma metastatic, Ewing’s tumor recurrent	pbi-shRNA-EWS/FLI1	cationic liposomes	*EWS/FLI1 fusion gene*	I	
NCT01505153	advanced cancer, metastatic cancer, solid tumors	pbi-shRNA-STMN1	lipoplexes	*STMN1*	I	

**Aptamers**						

NCT00881244	advanced solid tumors	AS1411	G-rich quartets + PEG	nucleolin	I	
NCT00512083	leukemia, myeloid	AS1411	G-rich quartets + PEG	nucleolin	II	
NCT00056199	Hippel-Lindau disease	EYE001	2′-OMe-modified purines and 2′-F-modified pyrimidines with 3′ inverted dT + PEG	VEGF	I	
NCT01486797	chronic lymphocytic leukemia	NOX-A12	PEGylated L-RNA (Spiegelmer)	CXCL12	II	Steurer et al.^57^
NCT01521533	multiple myeloma	NOX-A12	PEGylated L-RNA (Spiegelmer)	CXCL12	II	
NCT03168139	metastatic colorectal and pancreatic cancer	NOX-A12	PEGylated L-RNA (Spiegelmer)	CXCL12	I/II	

**DNAzymes**						

NCT01449942	nasopharyngeal cancer	DNAzyme targeting EBV-LMP1 (DZ1)	PS linkages	*EBV-LMP1*	I/II	Liao et al.^58^

**Transcription factor decoys**						

NCT00696176	head and neck cancer	STAT 3 DECOY	cyclic STAT3 decoy with hexaethylene glycol linkages	*STAT3*	I	Sen et al.^59^

Active or recruiting clinical trials						
Identifier	Disease	Oligonucleotide	Delivery strategies	Target	Phase	Reference
**Antisense ONs**						

NCT02598661	myelodysplastic syndromes	imetelstat^a^	PA linkages	RNA component of telomerase	II/III	
NCT04504669	clear cell renal cell cancer, non-small cell lung cancer, triple-negative breast neoplasms, squamous cell cancer of head and neck, small cell lung cancer, gastroesophageal cancer, melanoma, cervical cancer	AZD8701	cEt modifications with PS linkages	*FOXP3*	I	
NCT02499328	advanced solid tumors and metastatic squamous cell carcinoma of the head and neck	AZD9150	cEt modifications with PS linkages	*STAT3*	I/II	
NCT03334617	non-small cell lung cancer	AZD9150	cEt modifications with PS linkages	*STAT3*	II	
NCT02983578	advanced colorectal and lung non-small cell carcinoma, refractory colorectal, pancreatic, and lung carcinoma, stage II, III, IV pancreatic cancer AJCC v8, stage IIIA, IIIB, IIIC, IVA, IVB lung cancer AJCC v8, stage IIIA, IIIB, IIIC, IVA, IVB, IVC colorectal cancer AJCC v8	AZD9150	cEt modifications with PS linkages	*STAT3*	II	
NCT02546661	muscle-invasive bladder cancer	AZD9150	cEt modifications with PS linkages	*STAT3*	I	
NCT02781883	acute myeloid leukemia	BP1001	liposomes	*GRB2*	II	
NCT04196257	ovarian epithelial carcinoma, fallopian tube neoplasms, endometrial cancer, peritoneal cancer	BP1001-A	liposomes	*GRB2*	I	
NCT04072458	mantle cell lymphoma, peripheral T cell lymphoma, cutaneous T cell lymphoma, chronic lymphocytic leukemia, small lymphocytic lymphoma, follicular lymphoma, marginal zone lymphoma, Hodgkin lymphoma, Waldenström macroglobulinemia, diffuse large B cell lymphoma	BP1002	liposomes	*BCL-2*	I	
NCT05267899	advanced solid tumors	WGI-0301	LNP	*AKT1*	I	

**RNAi molecules**						

NCT04675996	solid tumor	miRNA-INT-1B3	LNP	*JNK1*	I	
NCT03819387	non-small cell lung cancer, pancreatic cancer, colorectal cancer	siRNA-NBF-006	LNP	*GSTP*	I	
NCT01591356	advanced malignant solid neoplasm	siRNA-EphA2	neutral liposomes	*EPHA2*	I	Wagner et al.^60^
NCT03608631	KRAS NP_004976.2:p.G12, metastatic pancreatic adenocarcinoma, pancreatic ductal adenocarcinoma, stage IV pancreatic cancer AJCC v8	siRNA-KRAS G12D	exosomes	*KRAS G12D*	I	
NCT04844983	squamous cell carcinoma *in situ*	siRNA-STP705	polypeptide nanoparticles	*TGF-β1* *COX-2*	II	
NCT04676633	hepatocellular carcinoma, liver metastases, cholangiocarcinoma	siRNA-STP705	polypeptide nanoparticles	*TGF-β1* *COX-2*	I	

**Small activating ribonucleic acids**						

NCT04710641	hepatocellular carcinoma	saRNA-MTL-CEBPA	liposomes	*CEBPA*	II	
NCT04105335	solid tumor, adult	saRNA-MTL-CEBPA	liposomes	*CEBPA*	I	
NCT02716012	hepatocellular carcinoma, liver cancer	saRNA-MTL-CEBPA	liposomes	*CEBPA*	I	Sarker et al.^61^

PS, phosphorothioate; 2′-OMe, 2′-O-methyl; 2′-O-MOE, 2′-O-methoxyethyl; cEt, 2′,4′-constrained ethyl; LNA, locked nucleic acid; miRNA, microRNA; siRNA, small interfering RNA; SNALP, stable nucleic acid lipid particles; TRiM, targeted RNAi molecule; SNA, spherical nucleic acid; pbi-shRNA, bifunctional expression vector plasmid DNA-bifunctional short hairpin RNA; G, guanine; PEG, polyethylene glycol; dT, deoxythymidine; PA, N-3′-phosphoramidate; LNP, lipid nanoparticle; saRNA, small activating RNA; TGFβ2, transforming growth factor beta 2; TP53, tumor protein p53; C-MYB, cellular-myeloblastosis oncogene; RNR, ribonucleotide reductase; HRAS, Harvey rat sarcoma viral oncogene homolog; PKCA, protein kinase C-alpha; CRAF, Raf-1 proto-oncogene; BCL-2, B cell lymphoma 2; XIAP, X-linked inhibitor of apoptosis protein; CLU, clusterin; HSP27, heat shock protein 27; EIF4E, eukaryotic translation initiation factor 4E; KRAS, Kirsten rat sarcoma viral oncogene homolog; AR, androgen receptor; STAT3, signal transducer and activator of transcription 3; YAP1, Yes1-associated transcriptional regulator; HIF-1A, hypoxia-inducible factor 1 subunit alpha; EGFR, epidermal growth factor receptor; GRB2, growth factor receptor-bound protein 2; VEGF, vascular endothelial growth factor A; KSP, kinesin spindle protein; PLK1, polo-like kinase 1; HIF-2A, hypoxia-inducible factor 2 subunit alpha; PKN3, protein kinase N3; BCL2L12, B cell lymphoma 2-like protein 12; KRAS G12D, Kirsten rat sarcoma viral oncogene homolog G12D; TGF-β1, transforming growth factor beta 1; COX-2, cyclooxygenase-2; EWS/FLI1, Ewing sarcoma breakpoint region 1/Friend leukemia integration 1; STMN1, stathmin 1; CXCL12, C-X-C motif chemokine ligand 12; EBV, Epstein-Barr virus; LMP1, latent membrane protein-1; FOXP3, forkhead box P3; AKT1, AKT serine/threonine kinase 1; JNK1, c-Jun N-terminal kinase 1; GSTP, glutathione S-transferase Pi 1; EPHA2, ephrin type-A receptor 2; CEBPA, CCAAT-enhancer binding protein alpha. Cited from http://www.clinicaltrials.gov (accessed on 30 July 2023).

a Special case ASO (instead of focusing on mRNA, its action is directed at the RNA component of the ribonucleoprotein known as telomerase).

## Therapeutic classes of ONs

The understanding of DNA’s role in heredity in 1944^62^ and the description of its helical structure in 1953^63^ provided the essential knowledge and tools to discover and utilize the properties of ONs for future oncological therapies. Thus, the primary mechanism of action of ONs is based on recognizing and binding to specific messenger RNA (mRNA) via Watson-Crick base pairing, leading to gene silencing, steric block, or modified splicing patterns.^64^ Alternatively, aptamers identify their targets (small molecules, peptides, and proteins)^65^ based on their unique three-dimensional structures.^66^ Below, a condensed overview of different types of therapeutic ONs (Table 1) and their mechanism of action (Figure 1) is presented.

### Antisense ONs

Since 1978, when Paul Zamecnik, the father of antisense ONs, and his colleague, Mary Stephenson, developed a 13-mer-oligodeoxynucleotide to inhibit Rous sarcoma virus replication and cell transformation in chicken embryos,^67^ the antisense technology has shown incredible capabilities as molecular tools for *in vivo* cellular regulation.^68^

Thus, ASOs are short, under 30 nucleotides, synthetic single-stranded RNA (ssRNA) or single-stranded DNA (ssDNA) molecules^69^^,^^70^ designed to have a sequence that complements their target, DNA or different types of RNA: precursor miRNA (pre-miRNA), mRNA, and long non-coding RNA (Table 1).^71^ Furthermore, ASOs can reach multiple cellular compartments, including both the cytoplasm and the nucleus (Figure 1).^72^ Based on their mechanism of action, ASOs can be divided into ribonuclease H-dependent (RNase H) ONs and steric blocker ONs (SBONs).^73^^,^^74^ RNase H-dependent ASOs are ssDNA-based ONs that produce a DNA/RNA duplex upon binding to their complementary site on a target mRNA. The newly formed duplex recruits the ubiquitous enzyme RNase H, which leads to RNA strand degradation. ASOs that work in conjunction with RNase H can significantly reduce the expression of targeted RNA, achieving a substantial downregulation of both mRNA and protein levels ranging from 80% to 95%.^74^ SBONs lack DNA bases in their composition and function by physically obstructing the splicing or protein translation processes^75^ after targeting the AUG initiation codon.^74^ Three generations of modified ASOs have been developed to improve specific aspects, such as target specificity and stability against enzymatic degradation.

The first-generation ASOs were developed by introducing backbone modifications to the phosphate group connecting the nucleotides. Therefore, several chemical groups such as sulfur, methyl, amine,^71^ acetate,^76^ and borane^77^ replaced the non-bridging oxygen atoms in the phosphodiester bond. The most used chemical modification introduced in ASOs was methylphosphonate in 1981,^78^ phosphorothioate (PS) in 1987,^79^ and phosphoroamidate in 1988.^80^ PS chemistry remains a crucial modification in contemporary ON drugs (Table 2), facilitating cellular uptake and providing protection against nuclease degradation, extending their half-life from minutes to days.^71^^,^^81^

Because of the broad non-specific effects typical of first-generation ASOs, efforts to increase specificity led to the development of a new generation. In the late 1980s,^82^^,^^83^ the sugar backbone was modified by the addition of alkyl groups at the 2′ position of the ribose. Over the years, various 2′-O modifications have been studied, but currently, 2′-O-methyl (2′-OMe) and 2′-O-methoxyethyl (2′-O-MOE) modifications are considered the standard.^84^ In contrast to first-generation ONs, these ASOs have lower toxicity and a stronger binding affinity to their targets.^85^

In the 1990s, several modifications to the sugar-phosphate backbone led to the development of the third-generation ASOs such as locked nucleic acid (LNA), peptide nucleic acid (PNA), and phosphoroamidate morpholino (PMO).^71^^,^^85^^,^^86^^,^^87^^,^^88^ LNAs utilize a methylene bridge linking the 2′-oxygen and 4′-carbon of ribose to enhance stability, binding affinity, and inhibit backbone hydrolysis through conformational constraint.^89^^,^^90^ PNAs are ONs characterized by the substitution of the phosphodiester backbone with a polyamide backbone, composed of repetitive units of N-(2-aminoethyl) glycine, wherein the nucleobases are linked by a methyl carbonyl linker.^91^ By substituting ribose rings with morpholino rings and phosphodiester bonds with phosphorodiamidate bonds, PMOs ensured high solubility in aqueous solution.^92^ PNAs and PMOs showed an increased resistance to nuclease activity as well as lower binding affinity to plasma proteins, which facilitates their elimination through urine.^9^^,^^71^^,^^90^ Moreover, to further increase their resistance against nuclease, novel versions of ASOs, such as 2′,4′-constrained MOE, and constrained ethyl (cEt) bicyclic nucleic acids combined features of second-generation 2′-O-MOE and third-generation LNA.^93^

Notably, an optimized variant of ASOs with regard to the binding affinity to targets and resistance to nuclease degradation is offered by gapmers (Table 1; Figure 1). Thus, they have a chimeric structure comprising a central short DNA region flanked by sequences of PS-modified ribonucleotides.^94^^,^^95^^,^^96^ The central DNA “gap” region can bind target transcripts via complementary base pairing, thus recruiting RNAse H to degrade the target RNA. In contrast, the flanking “wing” regions protect the molecule from nuclease degradation owing to the presence of 2′-O-MOE, 2′-OMe, LNA, or cEt modifications.^77^^,^^95^^,^^97^^,^^98^^,^^99^

### RNAi molecules

The challenges associated with targeting oncological markers using small molecular drugs, recombinant proteins, and monoclonal antibodies have led researchers and clinicians to explore RNAi as an alternative strategy for tumor-targeted therapies (Table 2). Since its discovery, RNAi has been defined as a mechanism of gene silencing (Table 1; Figure 1) using small RNAs, such as microRNA (miRNA), small interfering RNA (siRNA), short hairpin RNA (shRNA), or bifunctional short hairpin RNA (bi-shRNA) that target a wide range of protein-coding transcripts.^100^

Thus, miRNAs are endogenous ssRNAs (about 22 nucleotides) and siRNAs, shRNAs, and bi-shRNAs are exogenous, double-stranded RNAs (dsRNAs) comprising about 15–30 nucleotide pairs.^101^^,^^102^ Besides having a double-stranded stem, the shRNA molecule has a loop of at least 4 single-stranded nucleotides and a 3′ end dinucleotide overhang.^103^^,^^104^ All RNAi molecules employ cellular internal processing machinery to induce gene silencing.^105^ Being endogenous molecules, miRNAs, after their biogenesis as precursors, primary miRNA (pri-miRNA), are then processed into pre-miRNAs by the class 2 RNAse III enzyme called Drosha and transported from the nucleus to the cytosol via the exportin-5 protein. Herewith, pre-miRNAs, as well as exogenous, synthetic siRNAs, shRNAs, and bi-shRNAs undergo processing by the RNAse III enzyme Dicer, resulting in mature molecules that will undergo loading into the RNA-induced silencing complex (RISC), serving as the antisense guide for target recognition. Upon binding to a complementary mRNA target, the antisense guide initiates degradation via the AGO2 protein.^101^^,^^102^^,^^106^^,^^107^^,^^108^ RNAi molecules binding to the target is conditioned by the seed region (nucleotides 2–8) and supplementary region (nucleotides 13–16 of the miRNA’s 3′ region) that recognize mRNA.^109^^,^^110^ However, being a short sequence of nucleobases, seed sequences of RNAi molecules have the potential to bind to the 3′ UTRs of many different genes, leading to a mixture of on- and off-target effects.^6^

Nevertheless, as various types of cancers exhibit abnormal levels of miRNA expression as a consequence of gene alterations, abnormal transcriptional control, dysregulated epigenetic changes, and defects in miRNA biogenesis machinery^111^ several miRNA mimics and anti-miRNAs have been synthesized and tested in clinical trials (Table 2) as well as used to better understand their possible impact on cancer development. Besides miRNAs, siRNA stands out as the most promising for future medical applications due to its easy synthesis and efficacy in gene silencing, independent of genome integration. Its therapeutic index can be significantly increased after encapsulation in nanoscale delivery systems. Therefore, promising applications of siRNA have been explored in the management of breast,^112^ lung,^113^ brain,^114^ thyroid,^115^ and bladder cancers.^116^ Nevertheless, the major limitation of siRNA-based therapies is their short lifespan *in vivo*. Two strategies have emerged to address this issue: the addition of chemical modifications of the siRNAs and the utilization of shRNA, which can be processed intracellularly into siRNA.^117^ Multiple chemical modifications of the backbone have been proposed to increase siRNA efficiency in cancer treatments. Thus, as the 2′-OH group of the ribose plays an important role in RNA cleavage by endoribonucleases, substituting the hydrogen from this chemical group with methyl and methoxyethyl increased serum nuclease resistance of the siRNA.^118^^,^^119^^,^^120^ Moreover, the ribonuclease resistance has been further enhanced by substituting oxygen from the phosphate backbone with either sulfur, fluorine, or boron (clinical trial NCT04169711, Table 2).^120^ Notably, to increase the siRNAs’ and shRNAs’ efficacy as well as their lifespan *in vivo* for future clinical applications, bi-shRNA has been developed.^121^ To enhance knockdown potency, bi-shRNA utilizes two different shRNAs: one with mismatched guide and passenger strands for cleavage-independent RISC loading that induces the rapid inhibition of protein synthesis and the other with perfectly matched strands for cleavage-dependent RISC loading that is responsible for a delayed effect via mRNA cleavage and degradation.^122^^,^^123^ Thus, this approach enabled the administration of lower systemic doses and reduced off-target effects in comparison with other RNAi therapeutics.^123^

### Aptamers

Aptamers are a class of ONs often referred to as “chemical antibodies” in the literature.^124^ They are short ssDNA or ssRNA molecules, ranging from 20 to 60 nucleotides in length. These molecules adopt three-dimensional structures and demonstrated the capacity to bind with high affinity to target molecules^125^ (Table 1; Figure 1) through specific mechanisms dependent on their geometry, electrostatic interactions, van der Waals forces, and hydrogen bond formation.^8^^,^^126^ This improved property of the aptamers is conferred by the *in vitro* procedure of selecting these structures, known as the systematic evolution of ligands by exponential enrichment (SELEX).^127^ Owing to their high specificity, aptamers hold promise as agents against various targets in cancer therapy (Table 2), including extracellular ligands and cell surface proteins.^124^ Furthermore, unlike antibodies, aptamers are characterized by minimal immunogenicity, low molecular weight, stable structure, plasticity of chemical groups, and efficient and low-cost chemical synthesis.^128^^,^^129^

Nevertheless, aptamers present several challenges that need to be addressed, such as stability and high renal clearance. Various chemical modifications and technological advances are being explored to address these issues.^130^ Thus, 3′ end capping with biotin or inverted thymidine and several sugar ring substitutions at the 2′ position, such as 2′-fluoro, 2′-amino, and 2′-OMe, were found to be very effective in increasing their resistance to nuclease degradation.^130^^,^^131^^,^^132^ Moreover, the half-life and thermal stability of a linear aptamer can be enhanced by circularization using either chemical or enzymatic ligation processes. The chemical ligation method offers a more adaptable approach as diverse linking strategies can be employed. Nevertheless, this approach requires complex organic synthesis and may lead to the generation of harmful byproducts. Enzymatic circularization enables the utilization of natural nucleotides, thereby avoiding the toxicity associated with chemical alteration. It represents a quick and easy way to modify aptamers to enhance their stability and broaden their range of applications while exerting minimal influence on their folding and functionality. The most frequently utilized enzymatic ligation strategy involves the application of T4 ligase and CircLigase.^133^^,^^134^ However, enzymatic reactions have a relatively low circularization yield due to their low selectivity for intramolecular circularization over intermolecular ligation.^135^ An alternative approach to circularizing ssDNA molecules through ligase-mediated ligation involves utilizing Twister ribozymes to flank the RNA of interest leading to cleavage followed by subsequent ligation of both ends by endogenous RNA ligase RtcB.^136^ Multimerizing individual aptamers or conjugating them with bulky moieties increases their size, thereby overcoming rapid renal filtration and prolonging circulation time.^137^ Also, linking cholesterol, dialkyl lipids, or polyethylene glycol (PEG) to aptamers improved their serum half-life and nuclease resistance.^138^^,^^139^^,^^140^ To enhance the binding affinity, base modifications with naphthyl, triptamino, isobutyl, and benzyl groups can be employed.^141^^,^^142^ For example, 5-(N-benzylcarboxyamide)-2-deoxyuridine modification of the AS1411 aptamer selectively increased its target affinity to cancer cells (clinical studies NCT00881244 and NCT00512083, Table 2).

### DNAzymes

DNAzymes are specific short (15–40 nucleotides) ssDNA sequences with catalytic activity.^143^ Scientists have used *in vitro* selection strategies to identify DNAzymes capable of catalyzing RNA cleavage, RNA and DNA ligation, and covalent modifications of nucleic acid substrates.^144^ Similar to SELEX, a nucleotide library is incubated with the substrate of interest to select the optimal DNAzymes with the required activity, affinity, and specificity. RNA-cleaving DNAzymes are the most studied DNAzymes in cancer research due to their gene silencing potential. In the presence of specific metal ions such as Mg^2+^, Pb^2+^, Mn^2+^, Cu^2+^, and Na^+^, DNAzymes can cleave the target mRNA (Table 1; Figure 1) by catalyzing the hydrolysis of the phosphodiester bond.^145^ Despite promising results *in vitro*, further research on DNAzymes as gene silencing agents revealed that their *in vivo* efficacy is limited by reduced catalytic activity caused by the poor availability of metal ions under physiological conditions.^146^^,^^147^ Hence, only one clinical trial has been completed for DNAzymes in oncology thus far (Table 2).

### TFDs

TFDs are small dsDNA fragments designed to mimic the precise binding site of a target transcription factor involved in cancer development (Table 1; Figure 1).^148^^,^^149^ After cell internalization, TFDs can effectively disrupt the abnormal expression of multiple disease-associated genes by selectively binding to specific transcription factors responsible for regulating the expression of these genes.^150^ Most of the clinical investigations have focused on TFDs that target nuclear factor kappa-light-chain-enhancer of activated B cells and signal transducer and activator of transcription 3, two transcription factors implicated in carcinogenesis, tumor progression, and drug resistance in many types of cancers (Table 2).^151^^,^^152^

### sgRNA

First described as an “adaptive immune system” in bacteria and archaea to safeguard against viruses,^153^ the CRISPR-Cas system proved to be an efficient tool in cancer drug development due to its ability to precisely cleave and target multiple genomic regions associated with cellular malignant transformation.^154^ Thus, this technology facilitates the correction of genomic errors, regulation of gene expression, and cost-effective manipulation of genes in cells (Table 1; Figure 1).^155^

The CRISPR-Cas system consists of two main components, an sgRNA molecule and the Cas9 nuclease, forming together a complex that can cleave specific DNA sites.^156^ The primary function of the sgRNA is to guide Cas9 endonuclease to a specific location within the genome, where it induces a double-stranded break in the DNA. The inherent DNA repair processes subsequently maintain genomic stability through two distinct pathways: an error-prone non-homologous end-joining method, which can result in deletions and insertions (indels), and a less-frequent homology-directed repair mechanism, which mends the DNA damage by incorporating external repair templates to the damaged area.^157^ The sgRNA is composed of two segments: a constant sequence that creates a scaffold by several stem-loops for binding the Cas9 nuclease and an adaptable 5′ end segment of 20 nucleotides that is altered to match the target DNA sequence, allowing customization to various targets.^156^

An alternative CRISPR-Cas technique employs a modified, catalytically inactive Cas9 enzyme, known as nuclease-dead Cas9 (dCas9) to either activate or repress targeted genes.^157^ dCas9 is an RNA-guided DNA binding protein generated by the inactivation of its two catalytic domains that are fused to transcription modulating domains.^158^ Then, gene-specific sgRNAs guide the dCas9-transcription modulating domain complex to effector domains of specific DNA sequences to either repress (CRISPRi) or activate (CRISPRa) the transcription of target genes. While CRISPRi possesses the capability to inhibit transcription by either directly obstructing RNA polymerase activity or employing effector domains (Krüppel-associated box domains), CRISPRa solely utilizes effector domains to induce transcriptional activation.^159^ Nuclease-dead Cas9 has the potential to advance research on the biological functions of various genes in cancer.^160^

## Necessity for tumor-targeted delivery of ONs

**Figure 2:**
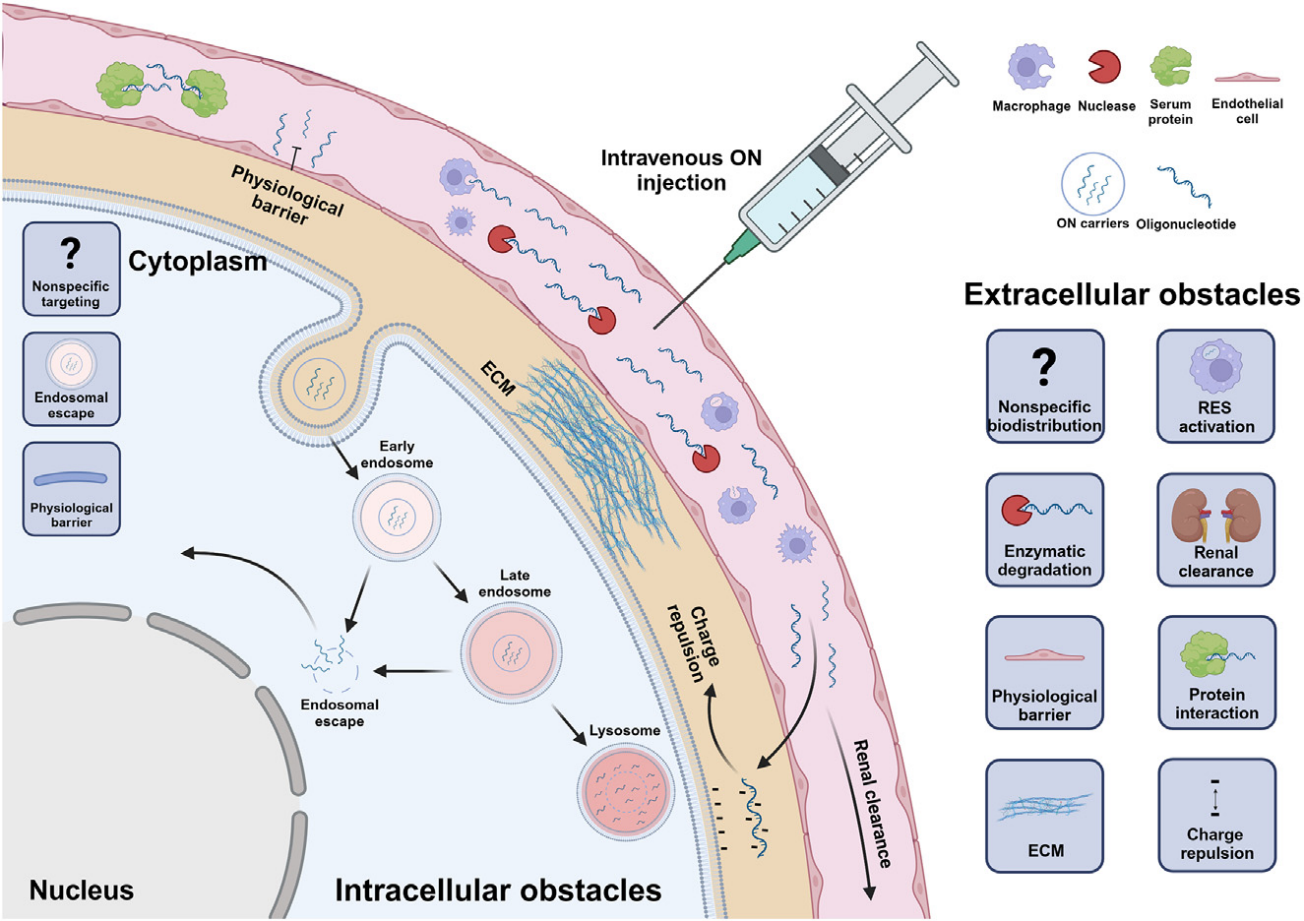
Extracellular and intracellular challenges of *in vivo* delivery of ONs Systemic administration of naked ON drugs often results in inadequate targeting of the specific tissue or cells, leading to non-specific distribution. Only a small ON fraction evades macrophage uptake, nuclease degradation, renal clearance, and serum protein adsorption. In addition, physiological barriers such as endothelium, cell membranes, nuclear membranes, or extracellular matrix (ECM) impede ONs from reaching their therapeutic target. Following cellular uptake, ONs must bypass endosomal entrapment to reach the target. A large proportion of the originally administered ON drugs does not effectively reach its final target. Created with BioRender.com.

Cancer therapies based on naked ONs have significant limitations regarding their therapeutic index, which depends on their ability to overcome different biological barriers associated with specific administration routes in the human body (Figure 2). Thus, upon systemic administration, naked ONs encounter extracellular obstacles such as non-specific biodistribution, elimination through the reticuloendothelial system (RES), nuclease degradation in the serum, renal clearance, and specific organ barriers including endothelial and blood-brain barrier (BBB) or mucus barrier in the intestine (Figure 2).^161^^,^^162^

Oral administration of ONs might be a very convenient route for systemic delivery due to the ease of administration, different dosage possibilities, and no constraints regarding sterility, size, and charge of the formulation. However, this route is not currently used in the clinic as there are important barriers that hinder ON accumulation at the target tissue, such as degradation determined by low pH and gastrointestinal enzymes, mucus barrier, low permeability of the intestinal mucosa due to the tight junctions, macrophage clearance, and intestinal peristalsis.^163^ Different parenteral routes have been used for the administration of ONs in cancer therapy according to their pharmacokinetics as well as the targeted tissues. Among systemic delivery, subcutaneous and intravenous (i.v.) routes are the most used for ON administration in the clinic due to their rapid systemic distribution, especially i.v. injection. However, the half-life and bioavailability of the i.v. administered ONs could also be increased when their binding to plasma proteins is strong.^164^^,^^165^ Although systemic delivery favors the ONs accumulation at the site of action, a significant amount also reaches highly vascularized organs with fenestrated endothelia, such as the kidney, liver, and spleen, showing hepatotoxicity- and/or nephrotoxicity-associated risks.^162^^,^^166^^,^^167^ Moreover, ONs are small molecules (about 3–6 nm) that undergo kidney ultrafiltration, being rapidly cleared from the organism.^168^ Besides kidney clearance, the RES mononuclear phagocytes, including Kupffer cells and splenic macrophages, play a significant role in degrading naked ONs from the bloodstream.^169^

In addition to systemic administration, local administration routes of ONs to tumors have gained increased interest due to several advantages such as bypass of various organ anatomical barriers, increased accumulation and retention time at the target site resulting in efficient uptake, fewer off-target effects, and reduced toxicity. Several local administration methods can be used to target ONs in different organs: intratumoral injection (direct injection or by using other platforms such as hydrogels), intrathecal injection, intraperitoneal injection, nose-to-brain administration, direct intravitreal injection, inhalation or intratracheal administration, etc.^170^^,^^171^^,^^172^ Although not currently used in the clinic, intratumor injection of ONs allows the achievement of high drug concentration *in situ* while using a lower dose, but it can be technically difficult in human patients.^173^^,^^174^ Moreover, the extracellular matrix is a dense barrier within tumors that significantly limits ON diffusion to deep tumor sites due to their macromolecular nature.^175^ Unlike healthy tissues, in tumors, the lymphatic drainage is hampered, with a leaky vasculature, leading to elevated interstitial fluid pressure that increases proportional to the distance from the vessel, hindering the homogeneous distribution of ONs throughout the tumor.^176^ In addition, tumor microenvironment conditions, such as low pH resulting from acid metabolite accumulation, can also contribute to ON delivery failure.^177^

Intrathecal and intravitreal injections are invasive and induce significant inflammation at the injection site. Similar to blood vessel endothelium, the BBB prevents ONs as well as other drugs from entering the brain parenchyma since endothelial cells are interconnected by tight junctions that provide a barrier function with a higher electrical resistance than peripheral capillaries, which prevents extravasation of molecules larger than 400 Da.^178^^,^^179^ Intraperitoneal administration of ON therapeutics is used to treat the tumors located in the peritoneal cavity or scattered throughout the peritoneum where systemic drug delivery is not successful. The main advantages of intraperitoneal administration of ONs are prolonged retention and the capability to administer large volumes of drug suspension.^180^

Besides extracellular barriers associated with different delivery routes significant obstacles given by the cellular structures of the target sites must be overcome to fulfill ONs’ pharmacological effects (Figure 2). Thus, intracellular challenges that most ONs encounter start with cellular uptake. Because of their negative charge, ONs are restricted from passing through the negatively charged cellular membrane due to electrostatic repulsion.^181^ In addition, altered membrane lipid structure and elevated cholesterol concentration make the tumor cell membrane less permeable.^182^ Upon internalization, successfully delivered ONs are often taken up into endosomes where they can be degraded by compartment-specific enzymes, impairing their escape to the cytoplasm.^183^^,^^184^ Within the cytoplasm, an important obstacle is the potential off-target effects caused by binding to unintended targets with similar sequences to the target RNA and proteins.^11^^,^^185^ In addition, cytosolic innate immune activation via pattern recognition receptors (Toll-like receptors [TLR 3, 7, 8], RIG-like receptors, STING) is often induced by ONs.^84^^,^^186^^,^^187^^,^^188^

## Targeted delivery of ONs to tumors

**Figure 3:**
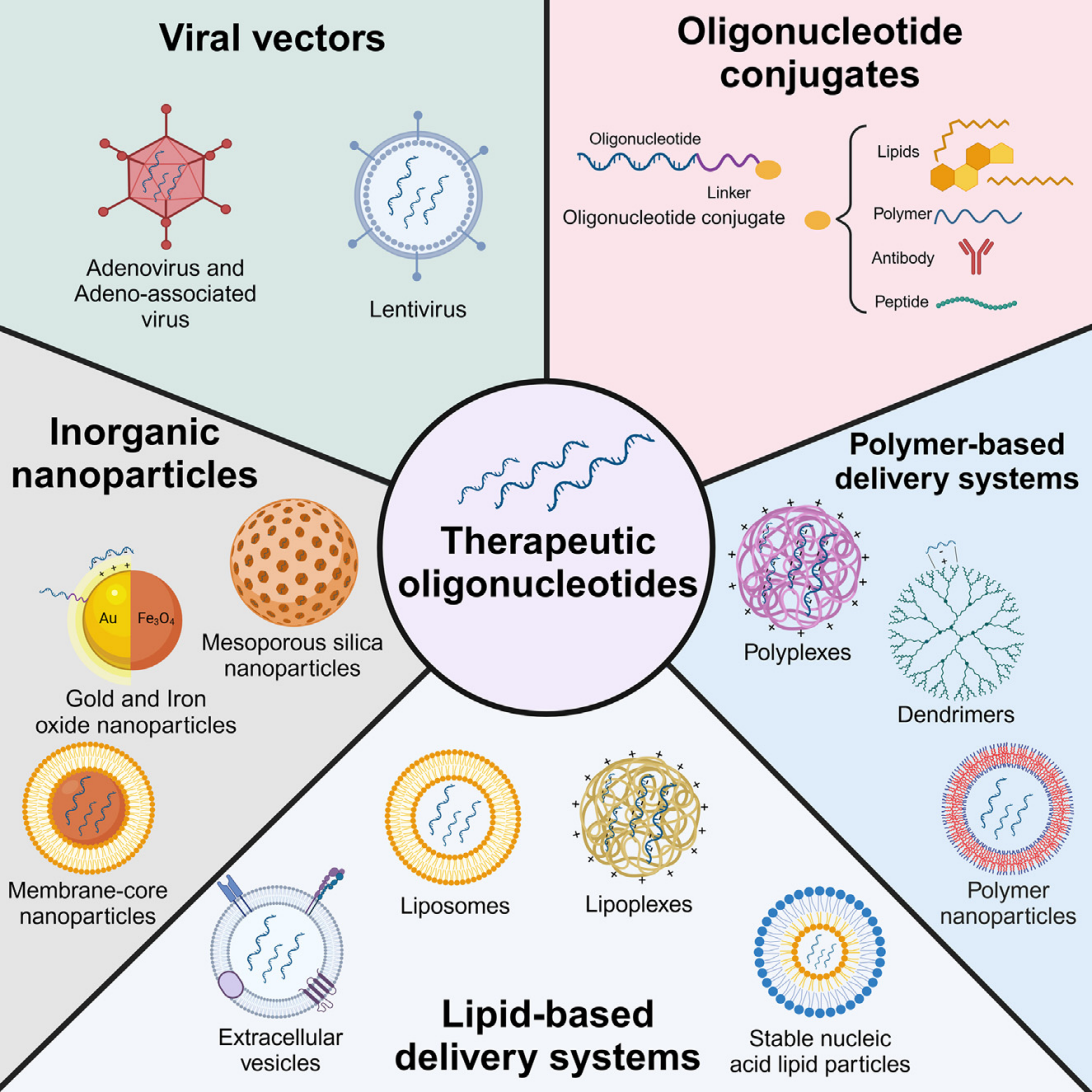
Viral and non-viral delivery systems for ONs in cancer Adenovirus, adeno-associated virus, and lentivirus are the most used viral delivery systems. Lipids, polymers, antibodies, and peptides are used for covalent conjugation to ONs for passive and active targeting. Polymer nanoparticles, dendrimers, lipid-based nano-carriers (liposomes, stable nucleic acid lipid particles, and extracellular vesicles), and inorganic nanoparticles (mesoporous silica and membrane-core nanoparticles) are the main non-viral vectors used to encapsulate ONs. Polyplexes, dendrimers, lipoplexes, and gold and iron oxide nanoparticles bind negatively charged ONs through electrostatic interactions. Created with BioRender.com.

Tumor-targeted delivery of ONs might efficiently counteract the abovementioned limitations and significantly improve their bioavailability and therapeutic efficacy, avoiding side effects on healthy cells. Therefore, the incorporation of ONs in viral and non-viral delivery systems ensures protection from endonuclease degradation and enhances cellular uptake, inducing their endosomal escape.^189^ The present section provides an overview of the main advantages and disadvantages of these potential delivery systems (Figure 3).

### Viral vectors

Since the first successful gene therapy in humans in 1992, when a retroviral recombinant virus was used to deliver adenosine deaminase gene to T cells, several viral vector-based therapies have been developed for the treatment of different genetic diseases and cancer.^190^ Viral vectors represent efficient delivery vehicles as they can transduce human cells and transfer genetic material, allowing for short- and long-term gene expression.^191^

To overcome cancer-related challenges, several viral vectors including adenoviruses (Advs), lentiviruses, and adeno-associated viruses (AAVs) have been the most used (Figure 3).^192^ Advs are icosahedral viruses characterized by a dsDNA structure and a diameter ranging from 90 to 100 nm. These viruses not only possess the capability to transport ONs but also activate the complement system and Toll-like receptors through their capsid and nucleic acid components, boosting intratumoral immune responses.^193^ AAVs, measuring 25 nm, are relevant in clinical trials due to lack of human pathogenicity, non-toxicity, and tissue tropism.^194^^,^^195^ Advs or AAVs can provide almost 100% transduction efficiency without integrating into the host genome, making them suitable for transient gene knockdown.^196^ Lentiviruses are enveloped, spherical retroviruses measuring approximately 100 nm in diameter with exposed glycoprotein that defines its tropism.^195^^,^^197^ One of the advantages of lentiviruses is the ability to perform a stable gene integration with a lower transduction efficiency but for a longer-term gene knockdown.^196^

In oncology, viral vectors are mainly used as gene delivery platforms in virus-based cancer vaccines, chimeric antigen receptor T cell as well as targeted oncolytic therapies.^198^ ONs such as siRNA and ASO became increasingly important in cancer treatment due to advances in their chemical modifications that were translated into increased stability. A great number of delivery methods to targeted cells have been developed, but their safety and efficacy need to be improved. Several preclinical studies used virus-based vectors to deliver different types of ONs to cancer cells.^199^^,^^200^ For example, a retroviral vector expressing siRNA targeting the mutant Kirsten rat sarcoma viral oncogene homolog (KRASV12) allele was shown to efficiently induce knockdown of the gene in the pancreatic cancer CAPAN-1 cells. In addition, the retroviral vector fully inhibited the tumorigenic capacity of the same cell line *in vivo*.^201^ Similarly, a recombinant adenovirus was used to transfer siRNA specific for survivin in different cancer cell lines and efficiently induced caspase-mediated apoptosis.^202^

Although the safety of the viral vectors used as delivery platforms has experienced significant improvements and they have several advantages over other delivery systems, viral vectors are not currently used in clinic to deliver ONs. Their use was limited mainly due to potential mutation risks, inflammation, and immunogenicity concerns. Non-viral vectors are considered safer and more efficient in delivering ONs to different targets.^189^

Other ONs, such as aptamers, were conjugated to a chemically modified AAV to target it to MCF-7 breast cancer cells, A549 lung carcinoma epithelial cells, and HeLa cells. Aptamer-conjugated vectors showed a 3- to 9-fold increase in transduction compared with non-conjugated vectors. In addition, *in vivo* studies showed that the DNA aptamer-virus conjugate showed no off-target effects.^203^

#### Non-viral delivery systems

Non-viral delivery systems for ONs have developed as a promising alternative to viral vectors by offering a safer and more efficient approach to delivering ONs to target cells. Moreover, non-viral systems have emerged to counteract the limitations of therapies based on ONs, such as lack of specificity for tumor tissue, physico-chemical properties (size, charge), as well as interaction with several biological barriers of the human body, including nuclease degradation, immune system, and quick clearance.^204^ These systems consist of various nanostructures, including polymer and lipid-based carriers, conjugates with cell-penetrating peptides and antibodies, and inorganic carriers (Figure 3).^189^^,^^205^

Therefore, tumor-targeted delivery of ONs via polymeric carriers (Figure 3) greatly increases their effectiveness and cellular bioavailability and overcomes the physical and biological barriers shown above.^204^^,^^206^^,^^207^^,^^208^^,^^209^^,^^210^^,^^211^ A great number of studies proved that polymer-based delivery systems, such as polymeric nanoparticles, dendrimers, and polymeric conjugates, significantly enhance the pharmacokinetic profile and local controlled release of ONs.^212^^,^^213^^,^^214^^,^^215^ Furthermore, delivery systems based on positively charged polymers (polyethyleneimine [PEI], chitosan, carbosilane, polyamidoamine, polypropylenimine) possess high encapsulation efficiency, enabling high ON concentrations accumulation in the tumor microenvironment. However, the significant toxicity and immunogenicity of the cationic polymers have limited their clinical use. Therefore, strategies such as nanostructures conjugation with PEG and hydrophobic moieties (e.g., cholesterol) have been explored (Figure 3).^215^^,^^216^^,^^217^^,^^218^^,^^219^^,^^220^^,^^221^^,^^222^ In addition to these advantages, due to their broad capacity of functionalization, polymeric nanosystems can offer protection against nuclease degradation and mediate endosomal escape and efficient cytosolic delivery of ONs in human ovarian and breast cancer cells.^210^^,^^211^^,^^218^^,^^223^^,^^224^^,^^225^ Moreover, polymeric conjugation with several chemical structures (cyclodextrins, PEG, folate) and/or association with physical factors enabled the development of the therapeutic platforms to simultaneously deliver siRNAs or sgRNA-Cas9 systems with cytotoxic drugs (docetaxel, paclitaxel, doxorubicin) and finally to induce synergistic antitumor effects on various tumor models.^218^^,^^219^^,^^220^^,^^223^^,^^226^^,^^227^^,^^228^^,^^229^ To enhance the specificity and selectivity of ONs for tumors, the conjugation of either nanostructure-based systems or ONs with targeting ligands, such as peptides or antibodies (Figure 3),^189^ advanced tumor-targeted therapies increasing their precise delivery into the tumor cells and even stromal cells.^230^^,^^231^^,^^232^^,^^233^^,^^234^^,^^235^^,^^236^^,^^237^ Notably, the development of a dual-targeting drug delivery system named Pep-21, combining a PD-L1-binding peptide with anti-miR-21 inhibitor, demonstrated the efficient binding to tumor cells and macrophages inducing decrease of miR-21 levels, tumor cell migration, and a macrophage polarization toward M1-phenotype and finally suppression of B16 melanoma progression.^236^ Furthermore, synthetic chimeric biomolecules such as antibody-ON conjugates (AOCs) take advantage of both the targeting capabilities of antibodies and the functional specificity provided by ON components. Several AOCs proved to be efficient in triggering receptor-mediated endocytosis upon binding to membrane receptor antigens. Nevertheless, the therapeutic efficacy of AOCs is dependent on several factors, including membrane antigen density, receptor turnover rate, and antibody-antigen affinity.^231^

Besides polymeric nanostructures, lipid carriers, including lipid-ON conjugates, liposomes, solid lipid nanoparticles, and extracellular vesicles (EVs) (Figure 3), possess unique characteristics and structures that enhance stabilization of ONs and their intracellular delivery.^189^^,^^238^^,^^239^^,^^240^^,^^241^^,^^242^^,^^243^^,^^244^^,^^245^^,^^246^ Liposomes are widely used as carriers for ONs due to their biodegradability, biocompatibility, and ease of formulation.^189^^,^^247^^,^^248^^,^^249^ Moreover, to improve targeting and selectivity for specific cells, the lipid bilayer can also be functionalized with ligands.^189^ In addition, different lipid types (cationic, anionic, neutral, and ionizable) from liposome composition make these carriers versatile platforms for ON delivery to tumors. Thus, cationic liposomes, often used for siRNA delivery due to their large cargo capacities, face challenges such as serum clearance and off-target effects that can be counteracted by strategies such as PEG conjugation.^250^^,^^251^ In a clinical trial, cationic liposomes carrying anti-vascular endothelial growth factor (VEGF) and anti-kinesin spindle protein (KSP) siRNA, known as ALN-VSP, showed an increased uptake in tumor cells and significant downregulation of VEGF and KSP levels when administered to patients with multiple types of cancer (Table 2).^51^ Moreover, liposomes made of ionizable lipids are able to induce destabilization of the endosomal membrane and efficient ON delivery into the cytosol.^252^^,^^253^^,^^254^^,^^255^ With this regard, Liu et al. developed a novel method to treat glioma cells by using hypoxia-responsive ionizable liposome-carrying anti-PLK1 siRNA that enhanced cellular uptake of siRNA, inducing a significant decrease in glioma cell proliferation both *in vitro* and *in vivo*.^256^ Nevertheless, latest studies suggested EVs as a better alternative for liposomes due to certain advantages over lipid nanoparticles.^257^ Thus, EVs, as intercellular communication tools, emerge as efficient vehicles for ONs due to their increased biocompatibility and targeting capacity, ability to cross biological membranes, and reduced immune stimulatory effects.^258^^,^^259^^,^^260^^,^^261^^,^^262^^,^^263^ Furthermore, a previous study demonstrated that the therapy based on fibroblast exosomes to transport anti-KRAS siRNAs eliminated metastatic pancreatic cancer in mice. Exosomes exhibited prolonged circulation time due to CD47-mediated immune evasion.^264^ Notably, these exosomes are also the subject of a phase I active clinical trial (NCT03608631) that focuses on the safety and optimal dosage of mesenchymal stromal cell-derived exosomes carrying anti-KrasG12D siRNA (iExosomes) in pancreatic cancer (Table 2).

Another strategy to deliver ONs to tumor tissues investigated the use of inorganic delivery systems (Figure 3) that offer advantages over lipid-based carriers due to their versatile functionalities and ease of synthesis with controllable size and surface characteristics.^265^ Gold nanoparticles (AuNPs), particularly, have gained a prominent role in oncology due to easy large-scale production, minimal size variations, and capacity to attach various ligands onto their surface, as well as *in vivo* biodistribution and excretion.^266^^,^^267^^,^^268^ Previous studies reported that AuNP conjugation with miRNA induced efficient knockdown in MM.1S multiple myeloma cells^269^ and AuNPs covalently linked to aptamer AS1411 improved the effects of imiquimod on HeLa cells and HEC-1-A human endometrial carcinoma cells.^270^ Besides AuNPs, iron oxide nanoparticles and mesoporous silica nanoparticles meet the prerequisites for effective ONs delivery by being non-cytotoxic, easily functionalized, and mediating high cellular uptake.^271^^,^^272^^,^^273^^,^^274^^,^^275^^,^^276^^,^^277^^,^^278^^,^^279^ Moreover, these inorganic nanoparticles conjugated with specific polymers (PEG, PEI) represent nanoplatforms for efficient intracellular delivery of ONs and cytotoxic drugs in various tumor models.^275^^,^^276^^,^^277^^,^^278^^,^^279^ Another strategy of siRNA delivery uses membrane-core nanoparticles that combine an inorganic nanocore functionalized to bind high amounts of siRNAs with an outer lipid bilayer derived from various cell membranes (e.g., red blood cells, platelets, white blood cells, cancer cells, stem cells, and bacteria) that protect nanoparticles from immune recognition, leading to optimal accumulation inside tumors.^280^^,^^281^^,^^282^^,^^283^ Thus, Chen et al. showed that HeLa cancer cell membrane-coated NPs could efficiently deliver a nanocore-loaded with doxorubicin and anti-PD-L1 siRNA, leading to suppression of PD-L1 and a stronger antitumor effect.^284^

### Clinical trials

Since 1998, when the first ON-based therapy (fomivirsen) was approved by the Food and Drug Administration (FDA) for the treatment of cytomegalovirus retinitis, several other ON-based therapies were approved by the FDA as well as the European Union’s European Medicines Agency (EMA) for the treatment of different diseases, other than cancer.^285^^,^^286^ In 2023, both the FDA and EMA approved two novel ON therapies for the treatment of geographic atrophy secondary to age-related macular degeneration (Iveric Bio) and amyotrophic lateral sclerosis (tofersen). Also, nedosiran (treatment of primary hyperoxaluria type 1) and eplontersen (treatment of the polyneuropathy of hereditary transthyretin-mediated amyloidosis in adults) were approved in 2023 but only by the FDA.^287^ We can see that the FDA generally approved new drugs in a shorter time compared with the EMA.^285^ Both the FDA and EMA have developed programs to allow faster approval of medicines with potential major public health interests. Therefore, in 2018, the FDA has initiated the Breakthrough Therapy and Fast Track designation programs, while the EMA introduced the PRIority Medicines designation plan. Nevertheless, there are several differences between the two agencies, including organization, advanced therapies classification, and clinical trial supervision. For example, the FDA has a broader classification of the gene therapy products compared with EMA.^288^

Despite this remarkable progress, the main limit to the widespread usage of ON therapeutics is their poor accumulation at the target tissue as a consequence of their enhanced clearance.^289^ Therefore, half of the ON-based therapeutics approved by the FDA have the liver as the target organ: inclisiran, lumasiran, givosiran, volanesorsen, patisiran, inotersen, defibrotide, mipomersen, and nedosiran.^290^ Yet, various strategies have been used to overcome this limitation. For example, the first ON-based therapeutic, fomivirsen, is locally administered by intravitreal injection and accumulates mainly in the retina and iris with minimal systemic exposure. Nusinersen, a splice-switching ON approved for the treatment of spinal muscular atrophy, is also locally administered by intrathecal injection, resulting in a good biodistribution. The most recently FDA-approved ON-based drug for the treatment of amyotrophic lateral sclerosis associated with a mutation in the superoxide dismutase 1 gene, tofersen, is also administered by the intrathecal route. On the other hand, ON drugs used for the treatment of Duchenne muscular dystrophy are systemically administered by i.v. injection, and it has been shown that they achieve the highest concentration in the kidneys.^285^ Another important characteristic of these successful ON therapies that can be exploited for the development of novel ON-based therapeutics for cancer treatment is the chemical modification of the nucleotides needed to increase their stability. For example, both the first and second generation of modifications such as PS, 2′-fluoro RNA, and 2′-OMe RNA were used in the development of several FDA-approved ON therapeutics: fomivirsen, pagaptanib, mipomersen, nusinersen, inotersen, givosiran, volanesorsen, lumasiran, and inclisiran. All the four ONs approved for the treatment of Duchenne muscular dystrophy have a phosphorodiamidate morpholino backbone. To further increase their chemical stability, accumulation at the target tissue and biodistribution, and to optimize the pharmacological effect, some of the FDA-approved ONs were delivered by different systems such as N-acetyl-galactosamine for givosiran, lumasiran, inclisiran, and vutrisiran, or lipid nanoparticles for patisiran.^291^ The latest siRNA-based therapeutic approved in 2023, nedosiran, uses Dicerna’s GalXC proprietary delivery platform consisting of N-acetyl-galactosamine sugars attached to the extended region of a dicer substrate siRNA molecule with a unique tetraloop configuration that ensures high stability and targeting to hepatocytes.^292^

As mentioned earlier, no ON-based therapy has been approved for cancer treatment by the end of 2023. However, there is an important number of ON-based therapies in clinical trials. Table 2 presents an overview of clinical studies investigating the potential of ONs as a promising therapeutic approach for various types of cancer treatment. These studies have explored different types of ONs, primarily focusing on chemically modified ASOs. In addition, miRNA and siRNA have been shown to be effective in several clinical studies. Among the most frequently targeted gene products presented in the table is B cell lymphoma 2 (BCL-2). These targets are being investigated for selective inhibition to suppress cancer cell growth, enhance apoptosis, and reduce tumor proliferation. Table 2 also provides information on the different phases of the clinical studies (phase I, II, and III) based on the specific target and delivery system being utilized. The phase of each study indicates its progress in the research process, ranging from early-stage exploratory trials (phase I) to larger-scale evaluations of efficacy and safety (phase III). A considerable number of clinical studies have reached the advanced stage of phase III, with many of them targeting BCL-2, clusterin, and protein kinase C-alpha. In contrast, a significant fraction of studies remain in the early stages of phase I, with only a limited number in phase II or I/II. Notably, all phase III studies employ ASO as the chosen ON platform. Other ONs, such as siRNA and miRNA, are predominantly found in the first, second, or first-to-second stage of development. As ongoing research advances, even though chemically modified ASOs remain at the forefront of investigational cancer therapies, miRNA and siRNA demonstrate promising potential for personalized cancer therapy.

## Conclusions and future perspectives

In the past few years, the research and development of ONs for cancer therapy have been fueled by the successful approval of several ON-based drugs for different non-cancer diseases as well as by the large-scale administration of mRNA-based COVID-19 vaccines. The development of ASOs, RNAi molecules, aptamers, DNAzymes, and TFDs has expanded the range of targets, especially for difficult or previously undruggable targets, making these ONs a promising class of biotherapeutics for a new era of anti-cancer therapies. However, the clinical use of ON-based therapeutics has been hindered by their high susceptibility to nuclease degradation, rapid blood clearance, immunogenicity, lack of inherent targeting mechanisms, low capability to cross physical and biological barriers, poor cellular uptake, and limited endosomal escape.

Numerous preclinical studies have been conducted to address the intracellular and extracellular challenges of naked ONs and to enhance their pharmacodynamic and pharmacokinetic properties for increased therapeutic efficacy against tumoral cells. Thus, experimental studies have demonstrated that chemical modifications, conjugation with different molecules, and utilization of nanoscale carriers can be employed to improve the delivery of ON-based therapeutics. However, further research is still required before the clinical translation of ONs can be fully realized.
